# Aldosterone does not require angiotensin II to activate NCC through a WNK4–SPAK–dependent pathway

**DOI:** 10.1007/s00424-012-1104-0

**Published:** 2012-05-03

**Authors:** Nils van der Lubbe, Christina H. Lim, Marcel E. Meima, Richard van Veghel, Lena Lindtoft Rosenbaek, Kerim Mutig, Alexander H. J. Danser, Robert A. Fenton, Robert Zietse, Ewout J. Hoorn

**Affiliations:** 1 Department of Internal Medicine, Erasmus Medical Center, P.O. Box 2040, Room D-405, 3000 CA Rotterdam, The Netherlands; 2 Water and Salt Research Institute, Department of Anatomy, Aarhus University, Aarhus, Denmark; 3 Institut für Vegetative Anatomie, Charité—Universitätsmedizin Berlin, Berlin, Germany

**Keywords:** Adrenalectomy, Aldosterone-sensitive distal nephron, Epithelial sodium channel, Sodium chloride cotransporter, SPAK

## Abstract

We and others have recently shown that angiotensin II can activate the sodium chloride cotransporter (NCC) through a WNK4–SPAK-dependent pathway. Because WNK4 was previously shown to be a negative regulator of NCC, it has been postulated that angiotensin II converts WNK4 to a positive regulator. Here, we ask whether aldosterone requires angiotensin II to activate NCC and if their effects are additive. To do so, we infused vehicle or aldosterone in adrenalectomized rats that also received the angiotensin receptor blocker losartan. In the presence of losartan, aldosterone was still capable of increasing total and phosphorylated NCC twofold to threefold. The kinases WNK4 and SPAK also increased with aldosterone and losartan. A dose-dependent relationship between aldosterone and NCC, SPAK, and WNK4 was identified, suggesting that these are aldosterone-sensitive proteins. As more functional evidence of increased NCC activity, we showed that rats receiving aldosterone and losartan had a significantly greater natriuretic response to hydrochlorothiazide than rats receiving losartan only. To study whether angiotensin II could have an additive effect, rats receiving aldosterone with losartan were compared with rats receiving aldosterone only. Rats receiving aldosterone only retained more sodium and had twofold to fourfold increase in phosphorylated NCC. Together, our results demonstrate that aldosterone does not require angiotensin II to activate NCC and that WNK4 appears to act as a positive regulator in this pathway. The additive effect of angiotensin II may favor electroneutral sodium reabsorption during hypovolemia and may contribute to hypertension in diseases with an activated renin–angiotensin–aldosterone system.

**Electronic supplementary material:**

The online version of this article (doi:10.1007/s00424-012-1104-0) contains supplementary material, which is available to authorized users.

## Introduction

Angiotensin II and aldosterone are the chief hormones of the renin–angiotensin–aldosterone system. In the last two decades, aldosterone has been recognized as the primary hormone regulating sodium transport along the distal nephron [13, 16, 44]. Aldosterone exerts its effects on three parts of the distal nephron, including the distal convoluted tubule (DCT), connecting tubule (CNT), and cortical collecting duct (CCD). In fact, this nephron segment is often referred to as the “aldosterone-sensitive distal nephron (ASDN)” [21, 39]. Via the mineralocorticoid receptor, aldosterone activates the two main sodium transporters in the ASDN: the thiazide-sensitive sodium chloride cotransporter (NCC) located in the “early” and “late” DCT [1] and the epithelial sodium channel (ENaC) located in the “late” DCT, CNT, and CCD [16]. Conversely, angiotensin II has traditionally been considered to act primarily in the proximal tubule, where it stimulates both the trafficking and phosphorylation of the sodium hydrogen exchanger type 3 [34]. The last few years, however, an emerging set of data has indicated that angiotensin II can also activate NCC [15, 35, 36, 42] and, to a lesser extent, ENaC [29]. These new insights have raised the question as to the respective roles of angiotensin II and aldosterone in the distal nephron and whether their effects are mutually dependent. This question is of both physiological and clinical relevance because aldosterone performs two completely different functions during hypovolemia (when angiotensin II is also increased) and hyperkalemia (when angiotensin II is not increased). This has been coined the “aldosterone paradox” [7, 9, 19, 43]. Of further interest is the recent discovery of a kinase network that can favor the sodium-retaining or potassium-secreting function of aldosterone by selectively activating ion channels. This kinase network consists of the Ste20-related kinase SPAK and several members of the WNK (With No K = lysine) kinase family [18, 19, 39]. Recently, we conducted a study addressing the independent role of angiotensin II in the distal nephron [42]. In adrenalectomized rats, we showed that angiotensin II induced phosphorylation of NCC in the absence of aldosterone. These effects were mediated by SPAK, which is capable of phosphorylating NCC [32]. Similarly, in oocytes and cells, San-Cristobal and colleagues showed that angiotensin II induced phosphorylation of NCC through WNK4 and SPAK [35]. Although WNK4 was previously shown to be a negative regulator of NCC [49], these authors proposed that angiotensin II may convert WNK4 to becoming a positive regulator of NCC [35]. Together, these recent data raise the question whether aldosterone requires angiotensin II to activate NCC and whether the effects of aldosterone and angiotensin II are additive. To address this, adrenalectomized rats were infused with vehicle, normal aldosterone, or high aldosterone in addition to the angiotensin II receptor blocker losartan.

## Materials and methods

### Animal studies

The animal protocol was approved by the Animal Care Committee of the Erasmus Medical Center (EUR 127-08-02). Three studies were performed in rats (all Sprague-Dawley, 15 weeks old, average weight 370 g; Charles River, Sulzfeld, Germany) (Supplemental Table 1). In the *first study*, all rats were adrenalectomized (via bilateral lumbodorsal incision) and were then randomized to receive no, normal (50 μg/kg/day), or high aldosterone (100 μg/kg/day). All rats also received losartan (10 mg/kg/day) [3, 25, 44]; this dose has been shown to inhibit 90 % of the angiotensin receptor type 1 receptors [53]. Blood pressure was measured every morning in conscious rats using a tail-cuff system after a 7-day acclimatization period (Kent Scientific Corporation, Torrington, CN, USA). In the *second study*, the sensitivity to hydrochlorothiazide or amiloride was tested, as described previously [14, 33]. Briefly, rats were adrenalectomized and randomized into six groups. All rats received losartan (10 mg/kg/day). The first three groups also received aldosterone (100 μg/kg/day) for 4 days after which hydrochlorothiazide (25 mg/kg), amiloride (2 mg/kg), or vehicle was injected intraperitoneally. The other three groups served as controls and received the same diuretics or vehicle, but no aldosterone. The diuretics were injected on the day of maximal sodium retention (day 4) and a timed urine was collected 5 h before and after these injections. Diuretic sensitivity was defined as the urine sodium to creatinine ratio. In the *third study*, the potentially additive effects of angiotensin II and aldosterone were tested. For this, samples from the first study and samples from our previously conducted study [42] were used. Four adrenalectomized rats receiving aldosterone (50 μg/kg/day) were compared to three adrenalectomized rats receiving aldosterone (50 μg/kg/day) and losartan (10 mg/kg/day). In all studies, animals were placed in metabolic cages after adrenalectomy and insertion of minipumps (Alzet, Cupertino, CA, USA). Throughout the study period, they were provided with normal rat chow and drinking fluid (0.9 % NaCl) ad libitum; normal saline was selected to compensate for natriuresis after adrenalectomy. All rats also received dexamethasone as glucocorticoid replacement (12 μg/kg/day) [38].

### Tissue preparation and plasma and urine measurements

Plasma renin activity and plasma aldosterone were measured as described previously [42]. Urine sodium and creatinine were determined with an automatic analyzer (Modular IPPE, Roche Diagnostics, Almere, The Netherlands). The right kidney was used for immunoblotting and was placed in an isolation buffer (10 mM triethanolamine, 250 mM sucrose, and protease inhibitors [Complete™, Roche Biochemicals, Indianapolis, IN, USA]) and homogenized. The whole kidney homogenate was subjected to differential centrifugation, as described previously [5]. Sixty microliters of both fractions was used for quantitative protein assay (Pierce, Thermo Scientific, Rockford, IL, USA) and the remaining samples were stored in 6× Laemmli at −80 °C for immunoblotting.

### Immunoblotting

Immunoblotting was performed as described previously [13]. For the first study, samples of all rats were immunoblotted simultaneously using two gels in one transfer apparatus including an internal standard. Antibodies against the following transport proteins were obtained: the α-, β-, and γ-subunits of ENaC (all 1:1,000), aquaporin-2 (AQP2) (1:1,000; all StressMarq, Victoria, BC, Canada), NCC and actin (1:500 and 1:100,000; Millipore, Temecula, CA, USA), SPAK (Cell Signaling, Boston MA, USA), and WNK4 (Division of Signal Transduction Therapy, University of Dundee, Dundee, Scotland, UK). Antibodies against pNCC (1:500) were generated by one of the investigators (RAF). Specificity of the SPAK antibody was confirmed using kidney tissue from SPAK^−/−^ mice (data not shown).

### Immunohistochemistry

The left kidney was used for immunohistochemistry. The midregion was sectioned into 2- to 3-mm transverse sections and immersion-fixed for an additional 1 h, followed by three times 10-min washes with 0.1 mol/l cacodylate buffer (pH 7.4). The tissue was dehydrated in graded alcohol, incubated overnight in xylene, and embedded in paraffin, and 2-μm sections were cut on a rotary microtome (Leica Microsystems, Herlev, Denmark). Immunolabeling was performed as described previously [12, 28]. Labeling was detected using a horseradish peroxidase-conjugated secondary antibody (Dako P448, goat anti-rabbit IgG, Glostrup, Denmark) and visualized with 0.05 % 3,3′-diaminobenzidine tetrachloride (Kemen Tek, Copenhagen, Denmark). Light microscopy was carried out with a Leica DMRE (Leica Microsystems, Herlev, Denmark).

### Quantitative PCR

Renal cortex was placed in RNALater (Qiagen, Valencia, CA, USA) and isolated using RNeasy® Mini Kit (Qiagen, Valencia, CA, USA). Five micrograms of RNA was used for first-strand cDNA (SuperScript™ II reverse transcriptase, Invitrogen, Carlsbad, CA, USA). The reaction was inactivated by raising the temperature to 70 °C for 5 min, followed by the addition of RNAse and 15 min incubation at 37 °C. The amplifications were performed using the SYBR Green PCR Master Mix (Applied Biosystem, Foster City, CA, USA). The reactions were set for 40 cycles at 60 °C in a Step One Plus System (Applied Biosystem, Foster City, CA, USA). Relative standard curve method was used for calculation. Standard curves enabled target gene quantification and normalization to an endogenous control (hypoxanthine–guanine phosphoribosyltransferase, Real Time Primers LLC, Elkins Park, PA, USA). All PCR products were checked by sequencing.

### Statistics

All data are expressed as the means and standard error of the mean. Group comparisons were made by using a Student’s  *t* test or analysis of variance with a post hoc test, as appropriate. Blood pressure data were analyzed using two-way analysis of variance. Correlations were calculated using Pearson’s rho. Because of the wide range, the natural logarithm of the plasma aldosterone concentration was used for these calculations. *P* ≤ 0.05 was considered statistically significant.

## Results

### Animal model to study the effects of aldosterone independent of angiotensin II

**Fig. 1:**
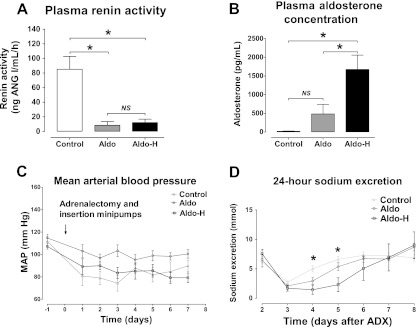
Physiological effects of aldosterone with losartan. Adrenalectomized rats received no aldosterone (*Control*, *n* = 5), normal aldosterone (*Aldo*, *n* = 5), or high aldosterone (*Aldo-H*, *n* = 5) with losartan for 8 days. At the end of the experiment, the plasma renin activity and the plasma aldosterone concentration were measured (**a**, **b**). During the experiment, arterial blood pressure and 24-h urinary sodium excretions were measured (**c**, **d**). By analysis of variance and post hoc test. *MAP* mean arterial pressure, *NS* not significant; *P < 0.01

Rats were adrenalectomized and then received the angiotensin receptor blocker losartan in addition to vehicle, normal aldosterone, or high aldosterone. This dose of losartan has been shown to inhibit 90 % of angiotensin type 1 receptors [53]. Plasma renin activity was significantly higher in the control group (Fig. 1a). The plasma aldosterone concentrations (Fig. 1b) confirmed that both the adrenalectomy and the delivery of two doses of exogenous aldosterone were successful. Blood pressure was similar in all three groups throughout the experiment (Fig. 1c). The aldosterone-infused groups retained more sodium, whose maximal effect was reached on the fourth day (Fig. 1d). At the end of the experiment, plasma creatinine and urine osmolarity were similar in all three groups (data not shown). Together, these data demonstrate that we successfully established an in vivo model to investigate the sodium-retaining effect of aldosterone independent of angiotensin II and independent of changes in blood pressure and glomerular filtration rate.

### Independent effects of aldosterone on transporters and regulatory proteins

**Fig. 2:**
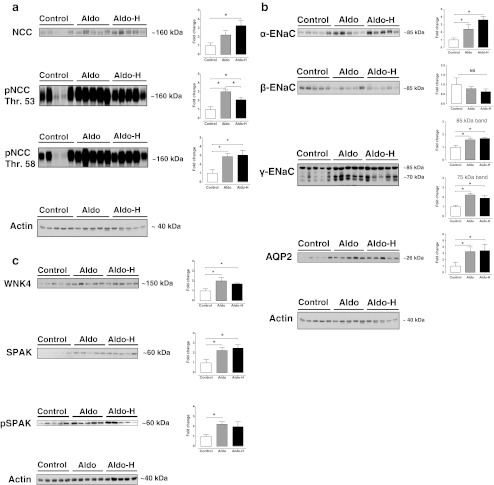
Effects of aldosterone with losartan on renal sodium transporters. Immunoblots showing the effects of aldosterone with losartan on the NCC (**a**), the ENaC and the water channel AQP2 (**b**). In addition, the effects of aldosterone with losartan on two regulatory kinases, WNK4 and SPAK, are shown (**c**). Whole kidney homogenates were differentially centrifuged to obtain plasma membrane fractions (used for all transport proteins) and intracellular fractions (used for the regulatory kinases). Densitometry was normalized for actin. *P < 0.05 by analysis of variance and post hoc test

**Fig. 3:**
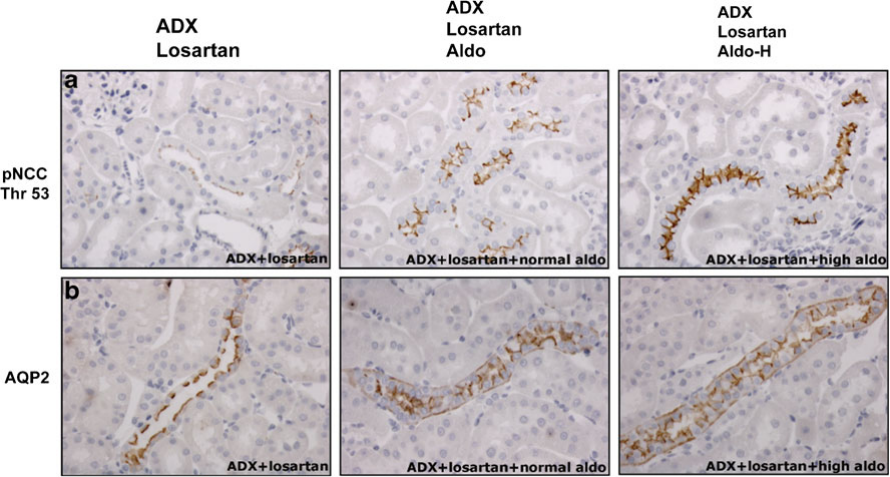
Immunohistochemical analysis of the NCC and AQP2. Immunohistochemistry for NCC phosphorylated at threonine 53 (pNCC) in the DCT (**a**) and the water channel AQP2 in the collecting duct (**b**). **a** and **b** show representative images of kidney sections from adrenalectomized rats treated with losartan only (*left*, ADX + Losartan), aldosterone and losartan (*middle*, ADX + Losartan + Aldo), and a high dose of aldosterone and losartan (*right*, ADX + Losartan + Aldo-H). In **a** and **b**, the more intense staining in the two experimental groups compared to the control group is clearly visible. In **b**, the more intense staining was attributed mainly to the induction of basolateral expression of AQP2

**Fig. 4:**
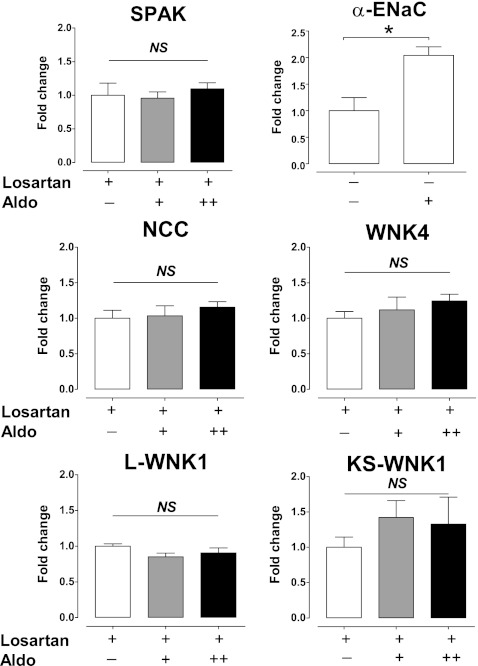
Effects of losartan with aldosterone on mRNA levels of NCC, α-ENaC, SPAK, and WNKs. The transcript abundances of SPAK, NCC, WNK4, L-WNK1, and KS-WNK1 are shown in adrenalectomized rats treated with losartan and no, normal, or high aldosterone. There were no significant changes among groups (using analysis of variance). As positive control, the transcript abundance of α-ENaC is shown for adrenalectomized rats that received no or normal aldosterone but no losartan (*upper right*). Aldosterone led to a significant increase in α-ENaC mRNA. All data represent the average value of five rats. In these experiments, results were normalized for the abundance of the housekeeping protein hypoxanthine–guanine phosphoribosyltransferase. As a control, specificity of the amplified products was determined using melting curve analysis and by product sequencing. *NS* not significant; *P < 0.01

Aldosterone infusion increased the abundance and phosphorylation of NCC twofold to threefold (Fig. 2a). A further increase in protein expression with the higher aldosterone dose was observed only for total NCC, but not for phosphorylation at threonine 53 and 58. Both doses of aldosterone also increased the α- and γ-subunits, but not the β-subunit of ENaC (Fig. 2b). The higher dose of aldosterone increased α-ENaC abundance from approximately twofold to fourfold, whereas the increase in γ-ENaC abundance was similar with the normal and high aldosterone doses. Both the 70- and 85-kD subunits of γ-ENaC increased significantly with aldosterone. The water channel AQP2 also increased approximately threefold with both doses of aldosterone (Fig. 2b). The abundances of the regulatory kinases WNK4 and SPAK increased with both aldosterone doses, whereas the phosphorylated form of SPAK increased significantly only with the normal aldosterone dose (Fig. 2c). Immunohistochemistry confirmed the increase of phosphorylated NCC at threonine 53 and of AQP2 (Fig. 3). It was of interest that aldosterone increased the total expression of AQP2 mainly by inducing basolateral expression. As well as protein expression data, we also studied the effects of aldosterone on the mRNA abundance of transporters and kinases. Although the mRNA abundance of α-ENaC increased significantly with aldosterone (Fig. 4), no significant changes in mRNA abundance were identified for NCC, SPAK, or WNKs. This could not be attributed to the administration of losartan because SPAK mRNA also remained unchanged with aldosterone alone (data not shown).

### Aldosterone increased the sensitivity to hydrochlorothiazide and amiloride

**Fig. 5:**
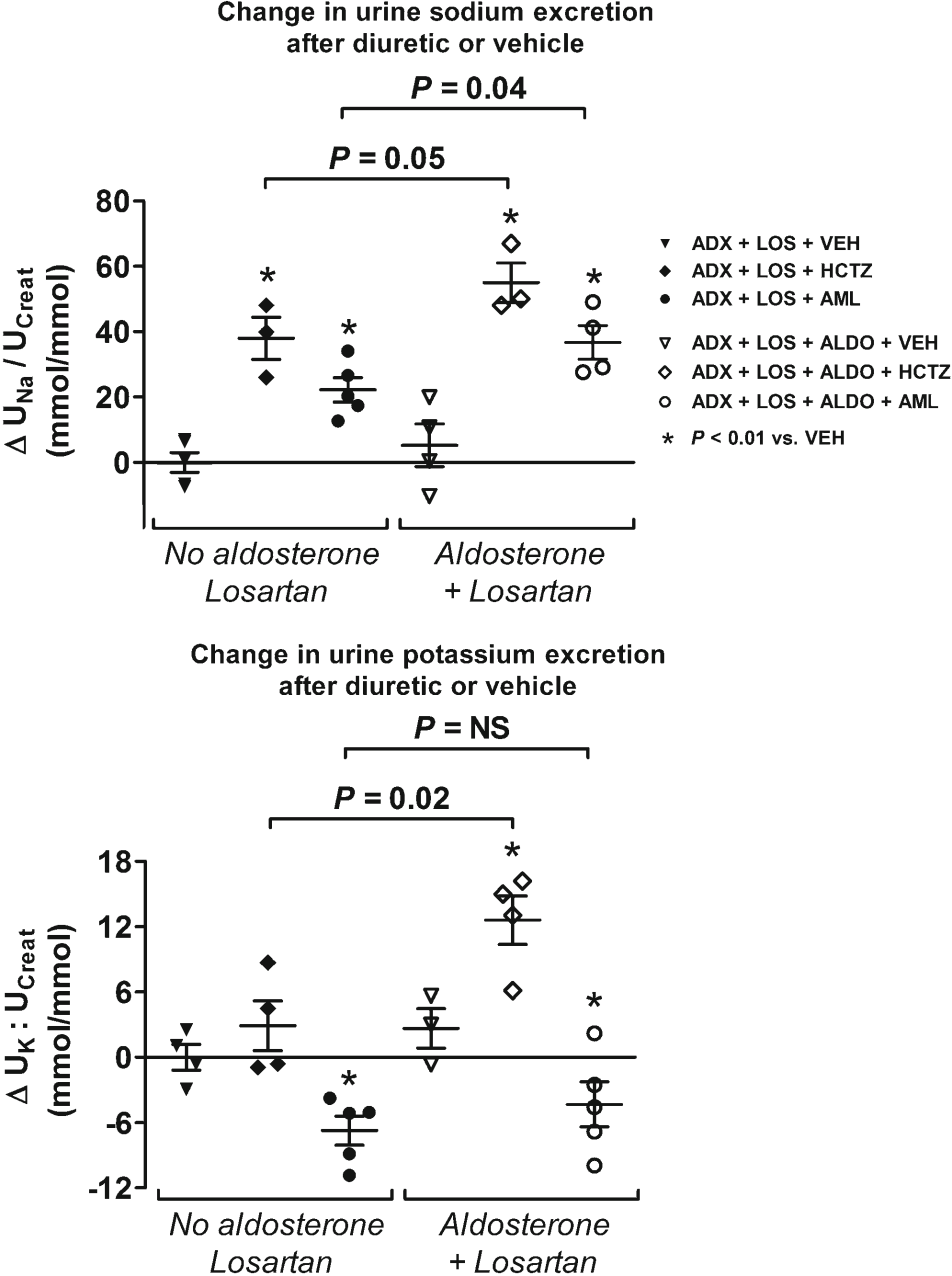
Diuretic sensitivity test. Results of a diuretic sensitivity test expressed as the change in urine sodium to creatinine ratio (∆*U*_Na_/*U*_Creat_) or urine potassium to creatinine ratio (∆*U*_K_/*U*_Creat_) before and after the injection of vehicle or diuretic. Each symbol represents one rat. The three groups in the left of the figure (*black symbols*) represent adrenalectomized rats (*ADX*) that received losartan (*LOS*). After 4 days, one of these groups was injected with vehicle (*VEH*, *black inverted triangle*), whereas the other two groups received a diuretic, including hydrochlorothiazide (*HCTZ*, *black diamond*) or amiloride (*AML*, *black circle*). The three groups in the right of the figure (*open symbols*) represent adrenalectomized rats that received aldosterone (*ALDO*) with losartan. After 4 days, one of these groups also received vehicle (*white inverted triangle*), whereas the other two groups received a diuretic, including HCTZ (*white diamond*) or AML (*white circle*). In all groups, diuretic treatment resulted in a significantly higher *U*_Na_/*U*_Creat_ than vehicle (*P < 0.01 using analysis of variance). *U*_K_/*U*_Creat_ increased significantly with HCTZ (except in the group without aldosterone) and decreased significantly with AML. The natriuretic and kaliuretic response to HCTZ in rats that also received aldosterone was significantly greater (*P* = 0.05 and *P* = 0.02, respectively). AML also caused a greater natriuretic response to HCTZ in rats receiving aldosterone (*P* = 0.04)

A diuretic sensitivity study was conducted in a separate experiment as a measure of the activity of NCC and ENaC during treatment with aldosterone and losartan (Fig. 5). Diuretic sensitivity was defined as the difference in urine sodium to creatinine or urine potassium to creatinine ratio before and after the administration of vehicle or diuretic. As expected, diuretic treatment resulted in significantly higher urine sodium to creatinine ratio in the groups with and without aldosterone. Hydrochlorothiazide increased kaliuresis, whereas amiloride reduced kaliuresis. More importantly, however, the increase in urine sodium to creatinine to hydrochlorothiazide or amiloride was significantly greater in those animals that also received aldosterone, suggesting increased activity of NCC and ENaC. Similarly, the increase in urine potassium to creatinine was also significantly greater in the animals receiving hydrochlorothiazide and aldosterone.

### Additive effect of angiotensin II and aldosterone

**Fig. 6:**
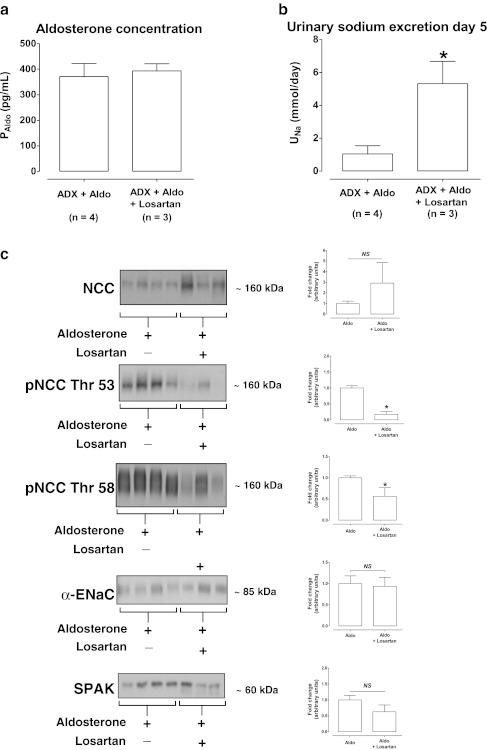
Additive effects of angiotensin II and aldosterone. **a** shows that seven adrenalectomized rats with similar plasma aldosterone concentrations were selected, three of which also received losartan. **b** shows that the addition of losartan resulted in a higher urinary sodium excretion. **c** shows that the addition of losartan reduced the phosphorylation of NCC, while there were no significant effects on total NCC, SPAK, and α-ENaC abundances. Densitometry was normalized for actin. *P < 0.05 by unpaired Student’s *t* tests. *ADX* adrenalectomy, *Aldo* aldosterone, *NS* not significant

In the final experiment, we examined whether aldosterone in combination with angiotensin II had an additive effect on renal sodium excretion and the abundance of NCC and ENaC. Seven rats were selected on the basis of similar plasma aldosterone concentrations (Fig. 6). Because only three of these rats also received losartan, this comparison allowed a selective analysis of an angiotensin II effect. Urinary sodium excretion was higher in adrenalectomized rats that received aldosterone and losartan, suggesting that inhibition of angiotensin II action resulted in greater natriuresis. Interestingly, phosphorylation of NCC was markedly reduced in the presence of losartan, both at threonine 53 and 58. Conversely, there was a trend towards a higher total NCC abundance with losartan, but this did not reach significance. Although a trend was observed for lower SPAK abundance with losartan, this was not statistically significant. Finally, the abundance of α-ENaC remained unchanged.

## Discussion

In addition to the classical role of aldosterone, there is an increasing body of evidence to suggest that angiotensin II also plays an important role in distal nephron sodium transport [15, 35, 36, 42]. Since both hormones are usually present together, it has been difficult to untangle their independent and potentially additive roles in distal nephron sodium transport. Here, we addressed this question by using an in vivo model of adrenalectomy and aldosterone with or without losartan. In the presence of losartan, aldosterone still caused renal sodium retention (Fig. 1), which was associated with increased expression of the distal sodium transporters NCC, pNCC, α-ENaC, and γ-ENaC (Figs. 2 and 3). Although two fixed doses of aldosterone were used, a range of plasma aldosterone concentrations were found. As a more functional measure of NCC and ENaC activity, we also showed that the natriuretic response to hydrochlorothiazide and amiloride was greater with aldosterone and losartan than with losartan alone (Fig. 5). This suggests that increased renal sodium retention with aldosterone and losartan was, at least in part, due to increased NCC and ENaC activity [33].

Next, we focused on the NCC regulatory proteins WNK4 and SPAK, the abundance of which increased with aldosterone and losartan (Fig. 2). Although SPAK has been clearly established as a kinase capable of phosphorylating NCC [22, 30], the role of WNK4 in the regulation of NCC is less clear (reviewed in [18]). Several studies have demonstrated that WNK4 can sometimes act as a negative regulator of NCC by diverting post-Golgi NCC to lysosomal degradation [40, 48–50]. However, WNK4 hypomorphic mice have reduced phosphorylation of NCC [27]. Similarly, WNK4 appears to mediate the phosphorylation of NCC during treatment with angiotensin II [35], insulin [37], cyclosporine [20], and tacrolimus [10]. Thus, the increase in both WNK4 and SPAK may have been related to their involvement in the phosphorylation of NCC, although more functional data are required to confirm this impression. The SPAK-mediated phosphorylation of NCC also suggests that transporter activity is regulated mainly by posttranscriptional mechanisms. This was further supported by the absence of changes in the mRNA abundance of either SPAK or NCC (Fig. 4). It is not unusual to see changes in protein expression without changes in mRNA levels. For example, in previous studies, the increase in NCC protein with aldosterone infusion [17] and the decrease in NCC protein during aldosterone escape [45] were not associated with corresponding changes in mRNA. While, like us, O’Reilly and colleagues did not detect differences in mRNA expression of long WNK1 (L-WNK1) or WNK4 in adrenalectomized mice treated with aldosterone for 6 days [26], they did observe an increase in kidney-specific WNK1 (KS-WNK1), while we observed a trend towards increased KS-WNK1 (Fig. 4).

Although SPAK, pNCC, NCC, and α-ENaC have all previously been shown to be aldosterone-sensitive [2, 13, 16, 42], this is the first in vivo model to demonstrate that aldosterone does not require angiotensin II for the upregulation of these proteins. Besides angiotensin II, vasopressin is of interest because it was recently shown to be capable of phosphorylating NCC through SPAK [23, 28]. Although we did not measure plasma vasopressin levels, the increase in AQP2 expression we observed (Figs. 2 and 3) might either have been indirect (through vasopressin) or direct (through aldosterone). Proof of the latter was provided by experiments in mpkCCDc14 cells, in which long-term incubation with aldosterone increased AQP2 protein abundance by increasing AQP2 mRNA translation [8]. As shown before [4], the increase in AQP2 was due mainly to increased *basolateral* expression of AQP2 (Fig. 3). It appears unlikely that the AQP2 translocation contributes to water movement because urine osmolality was unaffected and because AQP3 and AQP4 are also constitutively expressed in the basolateral plasma membrane [24]. Interestingly, high sodium intake by itself has also been shown to upregulate ENaC and AQP2 through an effect on collectrin, a homologue of angiotensin-converting enzyme 2 that is expressed in the apical membrane of the collecting duct [51].

Our final question was whether aldosterone and angiotensin II could have an additive effect on sodium transport in the distal nephron. To address this, we selected adrenalectomized and aldosterone-infused rats on the basis of similar plasma aldosterone concentrations (Fig. 6). Indeed, urinary sodium excretion increased with the addition of losartan to aldosterone-infused animals, suggesting a role of angiotensin II in renal sodium retention (Fig. 6). Immunoblot analysis suggested that phosphorylated NCC but not ENaC was involved in the additive effect of angiotensin II because aldosterone with losartan reduced the phosphorylation of NCC at threonine 53 and 58 (Fig. 6). This adds to recent work in which we show that angiotensin II induces phosphorylation of NCC independently of aldosterone [42]. The observation that angiotensin II selectively increases pNCC but not ENaC is likely to be of physiological importance, as it could help explain the “aldosterone paradox” [7, 9, 19, 43]. During hypovolemia, plasma levels of angiotensin II and aldosterone are elevated. On the basis of our data, this would favor sodium reabsorption by the DCT, limiting the flow and delivery to the CNT and CCD and, therefore, limiting potassium secretion [11]. Conversely, during hyperkalemia, when only aldosterone is elevated, sodium reabsorption by the CNT and CCD is more pronounced, stimulating potassium secretion. According to this model, angiotensin II could function as the “switch” between favoring electroneutral sodium reabsorption by the DCT and favoring electrogenic sodium reabsorption by the CNT and CCD [9, 46]. This model is further supported by the interesting recent finding that angiotensin II inhibits the renal outer medullary potassium channel (ROMK) [52]. However, the demonstration that a high potassium diet increased aldosterone but decreased NCC [6] suggests that other mechanisms are also involved. For example, a high potassium diet has been shown to increase the KS-WNK1/WNK1 ratio and the abundance of WNK4, which could inhibit NCC and activate ENaC and ROMK [9, 26].

A number of limitations of this study should be mentioned. First, the number of animals in some of the studies was small. Second, the results of our analysis on the additive effects of angiotensin II should be considered preliminary because samples from studies conducted at different times were compared. These results should, therefore, be confirmed in a separate study using a direct comparison (infusion of angiotensin II instead of losartan). Third, although not measured, the supplementation of aldosterone may have decreased plasma angiotensin II levels. Although losartan inhibits the angiotensin II type 1 receptor, it leaves the angiotensin II type 2 receptor unaffected. Therefore, in the control group, higher plasma levels of angiotensin II may have had effects through the angiotensin II type 2 receptor.

In this study, we focused on sodium transport by the aldosterone-sensitive distal nephron. Although this part of the kidney reabsorbs only 10–15 % of the filtered load of sodium chloride, altered function of this kidney segment can profoundly affect total body sodium and blood pressure [31]. This is illustrated by the fact that activating mutations of NCC and ENaC lead to renal sodium retention and hypertension [41, 47]. Clinically, the renin–angiotensin–aldosterone system is activated in many disease states, including several forms of hypertension, heart failure, liver cirrhosis, and nephrotic syndrome. Pharmacological inhibition of the renin–angiotensin–aldosterone system is the cornerstone of the treatment of these disorders. Unraveling the separate effects of angiotensin II and aldosterone is important when selecting appropriate pharmacological intervention for these diseases with angiotensin-converting enzyme inhibitors, angiotensin receptor blockers, or mineralocorticoid receptor blockers.

In summary, by using adrenalectomy, aldosterone, and losartan in rats, we have shown that aldosterone does not require angiotensin II to activate NCC through a WNK4–SPAK-dependent pathway. However, angiotensin II and aldosterone do appear to have additive effects on NCC. This explains a specialized system for the hormonal control of renal salt excretion that is relevant to health and disease.

## Electronic supplementary material

Below is the link to the electronic supplementary material.

Supplemental Table 1 (DOC 32 kb)
