# Supplementary material for: Aldosterone does not require angiotensin II to activate NCC through a WNK4–SPAK–dependent pathway
Source: Pflugers Arch. 2012 May 3;463(6):853–63. doi: 10.1007/s00424-012-1104-0 (PMC3350624; doi:10.1007/s00424-012-1104-0)
Supplement: Supplementary file 1 — (DOC 32 kb) [file 424_2012_1104_MOESM1_ESM.doc]

**Supplemental Table 1**

| **Study** | **Groups** | **No. of animals** | **Duration of study**  **(time of sample collection)** |
| --- | --- | --- | --- |
| 1 | ADX + Los | 5 | 8 days |
|  | ADX + Los + Aldo | 5 |  |
|  | ADX + Los + Aldo-H | 5 |  |
| 2* | ADX + Los + Veh | 4 | 4 days |
|  | ADX + Los + HCTZ | 4 |  |
|  | ADX + Los + AML | 5 |  |
|  | ADX + Los + Aldo + Veh | 4 |  |
|  | ADX + Los + Aldo + HCTZ | 4 |  |
|  | ADX + Los + Aldo + AML | 5 |  |
| 3 | ADX + Aldo | 4 | 8 days |
|  | ADX + Aldo + Los | 3 |  |

**Abbreviations:** ADX, adrenalectomy; AML, amiloride; Aldo, aldosterone; HCTZ, hydrochlorothiazide; Los, losartan; Veh, vehicle.

* The results for the urine sodium or potassium to creatinine ratios in Figure 5 sometimes show 3 or 4 observations; in these instances, there was either insufficient urine for measurement or a technical error during the measurement.
